# *In silico* ribozyme evolution in a metabolically coupled RNA population

**DOI:** 10.1186/s13062-015-0049-6

**Published:** 2015-05-27

**Authors:** Balázs Könnyű, András Szilágyi, Tamás Czárán

**Affiliations:** Department of Plant Systematics, Ecology and Theoretical Biology, Institute of Biology, Eötvös University, Budapest, Hungary; MTA-ELTE Theoretical Biology and Evolutionary Ecology Research Group, Budapest, Hungary; Parmenides Center for the Conceptual Foundations of Science, Munnich/Pullach, Germany

**Keywords:** RNA, folding, RNA-World, enzyme evolution, enzyme promiscuity, MCRS

## Abstract

**Background:**

The RNA World hypothesis offers a plausible bridge from no-life to life on prebiotic Earth, by assuming that RNA, the only known molecule type capable of playing genetic and catalytic roles at the same time, could have been the first evolvable entity on the evolutionary path to the first living cell. We have developed the Metabolically Coupled Replicator System (MCRS), a spatially explicit simulation modelling approach to prebiotic RNA-World evolution on mineral surfaces, in which we incorporate the most important experimental facts and theoretical considerations to comply with recent knowledge on RNA and prebiotic evolution. In this paper the MCRS model framework has been extended in order to investigate the dynamical and evolutionary consequences of adding an important physico-chemical detail, namely explicit replicator structure – nucleotide sequence and 2D folding calculated from thermodynamical criteria – and their possible mutational changes, to the assumptions of a previously less detailed toy model.

**Results:**

For each mutable nucleotide sequence the corresponding 2D folded structure with minimum free energy is calculated, which in turn is used to determine the fitness components (degradation rate, replicability and metabolic enzyme activity) of the replicator. We show that the community of such replicators providing the monomer supply for their own replication by evolving metabolic enzyme activities features an improved propensity for stable coexistence and structural adaptation. These evolutionary advantages are due to the *emergent uniformity of metabolic replicator fitnesses* imposed on the community by local group selection and attained through replicator trait convergence, i.e., the tendency of replicator lengths, ribozyme activities and population sizes to become similar between the coevolving replicator species that are otherwise both structurally and functionally different.

**Conclusions:**

In the most general terms it is the surprisingly high *extra viability* of the metabolic replicator system that the present model adds to the MCRS concept of the origin of life. Surface-bound, metabolically coupled RNA replicators tend to evolve different, enzymatically active sites within thermodynamically stable secondary structures, and the system as a whole evolves towards the robust coexistence of a complete set of such ribozymes driving the metabolism producing monomers for their own replication.

**Reviewers:**

This article was reviewed by Gáspár Jékely, Anthony Poole and Armen Mulkidjanian

## Background

The RNA-World scenario of the origin of life is based on the hypothesis that the first evolvable ancestors of all recent life on Earth must have been RNA-like macromolecules [1,2]. Even though we cannot (and probably will never be able to) positively prove this on the basis of fossil evidence, strong support to the prebiotic existence of an RNA-World comes from modern molecular biology: recent organisms still carry reliable clues suggesting that RNA had played a central role both in the metabolism and in the genetics of very early forms of life [3-5]. The fact that RNA enzymes occur in key positions of the molecular machinery of all living cells including the ribosome – the enzyme complex responsible for translation, of which RNA constitutes the functional core, is a convincing indirect evidence for an early RNA-dominated world of living organisms [6,7]. It took subsequent eons of evolution for the genetic role to be taken over by DNA and the metabolic role by protein enzymes [8] to yield the DNA-RNA-protein-World of life as we know it today.

The theoretical reason for the overwhelming majority of scientists studying the origin of life to accept the view that RNA (or some RNA-like replicator macromolecule, [9]) is the best candidate for the role of booting up life is the *dual nature* of RNA: it carries genetic information in its nucleotide sequence, and RNA molecules of different sequences are capable of catalysing a seemingly infinite variety of different chemical reactions [6,10-12]. Thus, prebiotic RNA enzymes (ribozymes) might have been *genes* and *enzymes* at the same time, so that two of the three essential functions of living systems (i.e., inheritance, metabolism and membrane, [13]) might have been embodied in the same chemical entity, comprising an infrabiological system [14] that is viable and evolvable in itself.

**Figure 1:**
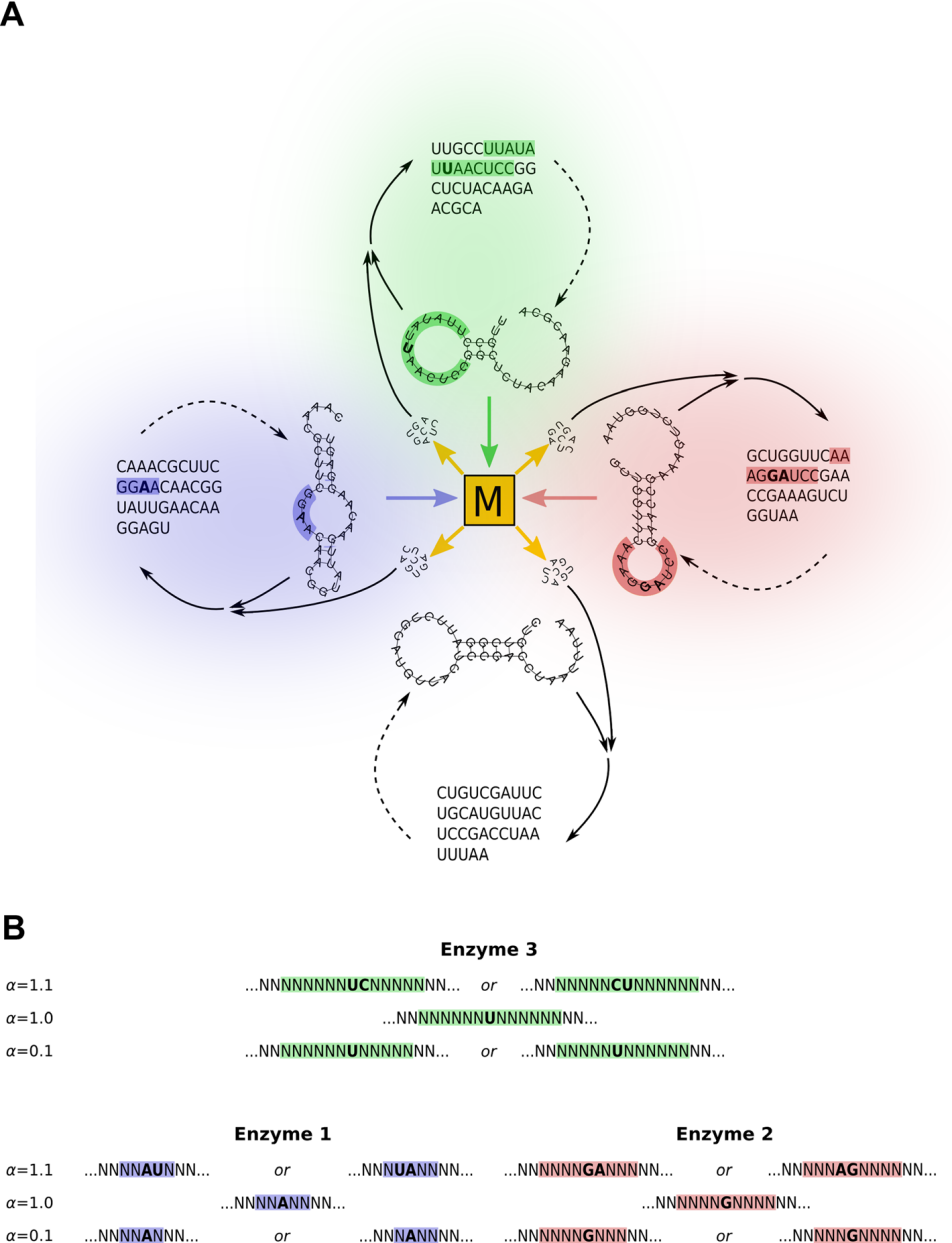
The Metabolically Coupled Replicator System with folded RNA molecules. **A**: The MCRS concept: *M* represents the hypothetical metabolic reaction network (metabolism). All reactions of *M* are catalysed by different replicators with enzymatic activities {1} (blue arrow), {2} (red arrow) and {3} (green arrow). The metabolism produces monomers (yellow arrows) which are the common resource of all replicator types (solid arrows). Each replicator is folded into 2D structures (dashed arrows) which may acquire catalytically active (coloured) regions including “active” and “helper” (boldface) bases. A parasitic sequence not contributing to monomer production (no arrow to *M*) but consuming monomers, is shown in white (no enzymatically active region). **B**: Colours represent catalitically active regions within a sequence; *α* is the catalytic efficiency of the given region (detailed explanation in text).

The *Metabolically Coupled Replicator System* (MCRS) is a family of computer models aimed at demonstrating that the RNA-World scenario is both ecologically and evolutionarily feasible under reasonable physical and chemical assumptions [15-19]. This means that – given the physico-chemical properties of RNA macromolecules and their precursors – a sufficiently diverse community of RNA populations can be maintained and evolved, in which the different RNA species cooperate to produce monomers for their own replication, and possibly also to supply other “common goods” for the replicator community (Figure 1A).

Even this early stage of prebiotic natural history must have been the result of an evolutionary process, because the first replicators had to be assembled without the help of other replicators, from chemical building blocks produced abiotically. Their lines of progeny must have evolved specific enzymatic activities through the process of *retroevolution* [20,21], driven by the self-inflicted selective (ecological) pressure due to the depletion of building blocks (monomers) in their environment. MCRS models address the evolution of prebiotic metabolism at this very early phase, assuming that a few terminal reactions of monomer production could be catalysed by some strains of replicators more effectively than they were supplied by the original abiotic source. The consequent fitness advantage of the cooperating replicators had set the retroevolutionary machinery in motion, to catalyse an expanding network of metabolic (or other beneficial) reactions.

The MCRS framework was originally developed as a toy model [15] in which all the physico-chemical attributes of the replicator molecules and their precursors (metabolites and monomers) were implicit in the updating rules of a stochastic cellular automaton. During the last 15 years we have refined the toy model approach in many ways [16,17], but the physico-chemically implicit nature of the MCRS framework has not been changed so far. The present study is the first one to consider explicit primary (nucleotide sequence) and secondary (2D folding) structures of the RNA molecules, thus taking the energetic and – to some extent – steric properties into account in both the genetic and the metabolic functions of the replicators. Another important novelty of this approach is that we consider mutations on the genetic (nucleotide sequence) level instead of the phenotypic mutations used in previous MCRS models [16,18]. The phenotypic effects of mutations are determined through consequential changes in the secondary structure of the mutant RNA molecule, which in turn determines its replicability, decay rate and enzymatic efficiency. The genotype-phenotype mapping is mediated by the minimum folding energy of sequences. We show that adding this realistic detail makes the MCRS approach even more robust than the toy model versions: the replicator system stays persistent and evolves to stationary states that are remarkably insensitive to changes in almost any model parameter.

## Methods

### Model framework

The model is implemented as a stochastic cellular automaton (SCA) on a rectangular grid of size *G × G*. The grid represents the mineral surface on which chemical reactions (replication and monomer production) occur. Each cell represents a discrete site on this surface that is either empty or binds one replicator molecule. Periodic boundary condition (toroidal surface) is used to avoid edge-effects. The model is initiated with a random population of replicators, occupying 80% of the sites at *t* = 0. The update process is random and asynchronous: the state of each site is updated at each time step in a random order. The number of update steps within one generation (from time step *t* to *t* + 1) is equal to the number of sites in the lattice. The lattice we used in all simulations consists of 300 × 300 sites (*G* = 300), thus one generation comprises 90.000 updates.

### Catalytic activities and metabolism

We assume that replicator molecules are RNA sequences with their secondary structures explicitly considered. Secondary structures and minimal free energies are calculated by the ViennaRNA Package 2.1.5 [22]. Secondary structure corresponds to enzymatic activity; energy determines the stability of folded RNA molecules. For sake of simplicity we assume that a sequence can be either in a folded state corresponding to the lowest free energy *E* (if there is an unique structure with optimal energy) or in a completely unfolded state with zero energy. The probability that a sequence is in the folded state is determined by Boltzmann statistics as follows:

1 $$ {p}_{\mathrm{fold}}=\frac{1}{1+{e}^{-cE}}, $$

where factor *c* scales the probability to yield *p*_fold_ ≈ 1 in case of optimal energy. Numerical experiments suggest that the optimal energy in the 15-75 nt range of sequence length has a lower bound at *E*_min_ = −25 kcal/mol. According to the probability distribution above a sequence with zero free energy is folded with probability 0.5, while a sequence of low energy is folded into (a stable) secondary structure with 0.5 < *p*_fold_ < 1.0. (At this stage we omit the possibility of folding into suboptimal structures.)

We assume that there are three well-defined secondary structures (active site configurations) corresponding to three different enzymatic activities (*α*_1_, *α*_2_ and *α*_3_) which are all required for metabolism to work. The structure-activity mapping follows the following simple rules (Figure 1B):

1. An internal loop (or bulge loop) of length five and a base *A* in its middle (3^rd^) position corresponds to the unit activity (*α*_1_ = 1) in the first enzymatic process. If there is a “helping base” *U* adjacent to this catalytic base *A*, then the activity is boosted by 10% (*α*_1_ = 1.1). If the loop is only four bases long but contains the catalytic base *A* in the 2^nd^ or 3^rd^ position, then the enzymatic activity, due to steric reasons, is only 0.1 (*α*_1_ = 0.1). In any other case there is no catalytic activity (*α*_1_ = 0).

2. A hairpin loop of length 9 and a base *G* in the middle (5^th^) position provides activity 1 (*α*_2_ = 1) for the second enzyme-catalysed process. Base *A* next to the catalytic base *G* increases the activity by 10% (*α*_2_ = 1.1). If the length of the loop is 8 (but contains the catalytic base *G* in position 4 or 5), then the activity is 0.1 (*α*_2_ = 0.1). In any other case there is no catalytic activity (*α*_2_ = 0).

3. A hairpin loop of length 13 and a base *U* in the middle (7^th^) position yields an activity of 1 (*α*_3_ = 1) in the third enzyme-catalysed process. Base *C* neighbouring catalytic base *U* increases the activity by 10% (*α*_3_ = 1.1). If the length of the loop is 12 (but contains *U* in positions 6 or 7) the activity is 0.1 (*α*_3_ = 0.1). In any other case there is no catalytic activity (*α*_3_ = 0).

An adequately long sequence can accommodate more than one active catalytic region (*promiscuous enzymes*). We assume that, due to steric reasons, a promiscuous enzyme cannot catalyse more than one reaction at the same time, thus we assume a sub-additive effect on different activities:

2 $$ {a}_i={p}_{\mathrm{fold}}\frac{\alpha_i}{m^{\sigma }}, $$

where *m* is the number of different active sites of a given molecule, and σ > 1 provides the sub-additive effect. With this factor we take into account the effect that for a promiscuous enzyme the necessary conformational changes for catalysis (“induced fit”) need more time than for mono-active enzymes because of the more rigid secondary structure. Relatively short sequences cannot act as promiscuous enzymes due to energetic constraints, however.

### Degradation, replication and mutation

In each time step, a replicator can replicate or decay. The probability of decay depends on the minimal free energy of the secondary structure (which is associated with its stability) according to the following equation:

3 $$ {p}_{\deg }=0.9-0.8\frac{E}{E_{\min }}, $$

where *E*_min_ is the optimal (minimal) free energy of the replicator in the relevant sequence length range. In the range of *L* = 10…70, *E*_min_ = −25 kcal/mol. The prefactor 0.9 is the maximum degradation rate corresponding to *E* ≈ 0. The minimum degradation rate is 0.1 (preventing unlimited longevity of sequences with optimal energy), which is ensured by the multiplier factor 0.8.

**Figure 2:**
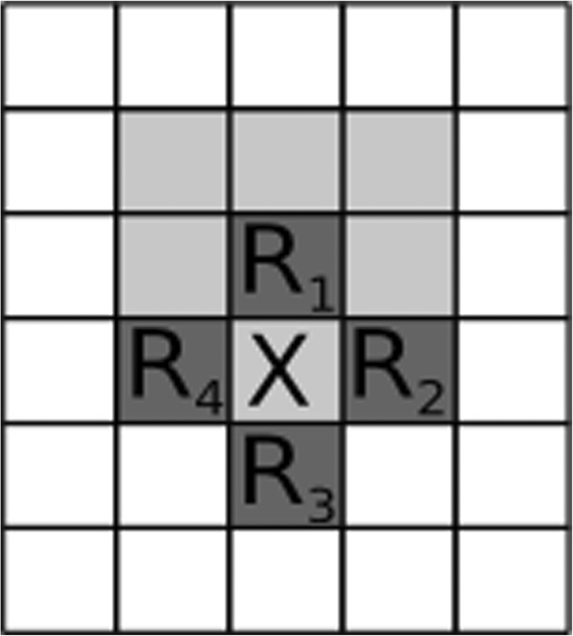
Neighbourhoods used in the model. Neighbourhoods defined in the model. Dark grey sites constitute the replication neighbourhood of the empty site X (von Neumann neighbourhood in this case). Light grey sites are the metabolic neighbourhood of replicator R_1_ (3×3 Moore neighbourhood). R_*i*_ (*i* = 1,2,3, 4) are replicators.

For a replication event to occur the focal replicator *s* must be complemented by all three different enzymatically active molecules in its metabolic neighbourhood (MET(*h,s*), the set of *h* sites concentric on the site of the focal replicator *s*). The metabolic activity (*M*_*s*_) around replicator *s* is the geometric mean of the different metabolic enzyme activities within its metabolic neighbourhood [15]:

4 $$ {M}_s=\sqrt[3]{{\displaystyle \prod_{j=1}^3{\displaystyle \sum_{i\in \mathrm{MET}\left(h,s\right)}{a}_{j,i},}}} $$

where *a*_*j,i*_ denotes the *j*^*th*^ type of activity (*j* = 1, 2, 3) of replicator *i* within the metabolic neighbourhood of size *h* around the focal replicator *s* (*i* ∈ MET(*h*, *s*)), see Figure 2 for an illustration of the metabolic neighbourhood. Due to the geometric mean any of the three types missing from the metabolic neighbourhood implies a total metabolic activity of zero (*M*_*s*_ = 0), and thus no chance for the focal replicator to be copied.

Besides the metabolic activity *M*_*s*_, the replication probability of a sequence *s* also depends on the minimal free energy of its secondary structure. Since replication is possible only in the unfolded state for any sequence, its replicability is proportional to the factor (1 − *p*_fold_). The length of the sequence also affects replicability through the time required for completing replication. In our simple model context the time consumption of a replication event is affected by two factors in a linear manner: the first one *b*_1_ is invariant (representing the length-independent initiation/termination steps of the replication process); the other one, *b*_2_, is proportional to the length *L* of the sequence (this is the replication process itself). The parameter *b*_2_ represents the time needed for the addition of a single nucleotide to the sequence being replicated. To sum up these two factors, the *replicability* (*R*) of a given replicator s is the following:

5 $$ {R}_s=g\frac{1}{b_1+{b}_2\cdot L}\left[l+\left(1-{p}_{\mathrm{fold}}\right)\right], $$

where *g* is a scaling factor and *l* (*l* > 0) ensures that even the best ribozymes (with *p*_fold_ close to 1) can replicate to some extent.

The claim *C*_*s*_ of replicator *s* belonging to the replication neighbourhood of an empty site to replicate into that empty site is the product of the metabolic activity (*M*_*s*_) and replicability (*R*_*s*_) of the replicator *s*:

6 $$ {C}_s={M}_s\cdot {R}_s. $$

The actual probability of template *s* to replicate into an empty site depends on its own claim and that of all the other replicators present in the replication neighbourhood of the empty site:

7 $$ {P}_s=\frac{C_i}{C_e+{\displaystyle \sum_l{C}_l}}, $$

where *l* runs through the (von Neumann-type) replication neighbourhood of the empty site (see Figure 2) and *C*_*e*_ is the claim of the empty site to remain empty. The probability that an empty site remains empty is ${P}_{\mathrm{empty}}=1-{\displaystyle \sum_l{P}_l}$. We set *C*_*e*_ to be an *ε* fraction of the theoretical maximum of the claims. To compute this maximum we assume that all metabolic neighbourhoods are optimal. Specifically, a focal replicator *s* has eight neighbours in its *h* = 3 × 3 metabolic neighbourhood and a single empty site into which it can place its copy. Thus the maximal metabolic activity in case of three different reactions requires 3, 3 and 2 replicators with optimal catalytic activity ($\overset{\wedge }{\alpha }=1.1$) each. We assume also that the active replicators are all monoactive, i.e., we disregard promiscuity. With this simplification we implicitly assume that all sequences are folded at the optimal energy: *p*_fold_ = 1. According to these postulates the theoretical maximum of metabolic activity is ${\overset{\wedge }{M}}_s=\sqrt[3]{{\left(3\overset{\wedge }{\alpha}\right)}^22\overset{\wedge }{\alpha }}=\sqrt[3]{18}\overset{\wedge }{\alpha }$. (Note that a common activity value *α* for all the three active centres implies a maximum metabolic activity of ${\overset{\wedge }{M}}_s=\sqrt[3]{18}\overset{\wedge }{\alpha }$) With this simplification the claim of the focal replicator is: $\overset{\wedge }{C}=g\frac{\sqrt[3]{18}}{b_1+{b}_2\cdot L}\overset{\wedge }{\alpha }$. With the default parameters (*g* = 10, *b*_1_ = 1, *b*_2_ = 0.05) and at *L* = 35 (in accordance with our numerical experience) the maximum claim is $\overset{\wedge }{C_s}=10.48$. As the claim of an empty site to remain empty is *ε* fraction of the maximum claim, $\overset{\wedge }{C_e}=1.048$.

Mutation takes place during a successful replication step with a per base probability: *p*_sub_, *p*_ins_, *p*_del_ of substitution, insertion and deletion, respectively. These three processes act independently.

### Diffusion

Empirical studies have proven that RNA precursors (intermediate metabolites and monomers) and RNA macromolecules (replicators) are capable of reversible binding to charged mineral surfaces, which implies two-dimensional diffusion of limited speed for small and large molecules [23,24].

### Diffusion of replicators

The speed of surface diffusion depends on the strength of interaction between the surface and the moving material. For example, the different diastereomers of sugars attach to the surface with different numbers of hydroxyl groups and consequently they move on it with different velocities, resulting in enantiomer selection [25]. For RNA molecules such simple relationships hold only if the sequence is short; longer RNA sequences always fold into complex three-dimensional structures which determine their speed of diffusion on the surface. Three-dimensional RNA structures cannot be reliably calculated yet, unfortunately, therefore we assume that different RNA molecules share the same diffusibility on average. With this simplifying assumption the movement of replicators on the surface can be implemented by the Toffoli-Margolus diffusion algorithm [26] which is scaled by a single parameter: the strength of diffusion (*D*). We used an asynchronous version of the original algorithm which was designed for synchronously updated simulations. The procedure is as follows: in each update step a randomly chosen 2 × 2 block is rotated by 90° clockwise or counter-clockwise with probability 0.5. The diffusion of replicators is scaled by parameter *D*, specifying the average number of such diffusion updates relative to one replication/decay update. Higher values of *D* correspond to higher diffusion rates. Note that *D* = 0 does not imply exactly zero diffusion, because a small non-zero rate of mixing comes from each new replicator being placed to a site adjacent to its template. Additionally, *D* = 1 means that each replicator moves on the grid surface four times on average during a generation time (from *t* to *t* + 1). The speed of replicator diffusion can be gradually decreased using *D* < 1 values, which means that the Toffoli-Margolus algorithm does not operate after every replication/decay update step. For example *D* = ½ means that the diffusion of replicators acts at every second update step on average.

### Diffusion of metabolites

The diffusion of RNA monomers and the metabolites along the chemical pathways producing them is implicitly considered in the size of the metabolic neighbourhood (*h*): larger *h* means faster metabolite diffusion, i.e., a longer distance that the metabolite (or monomer) molecule can cover on the mineral surface before being used in a reaction, degraded, or desorbed from the surface and lost to the third dimension. To simplify the treatment of metabolite diffusion, degradation and desorption we assume that all the metabolically vital enzyme functions have to be present within the metabolic neighbourhood of the replicator to be copied in order to supply monomers for its replication (Figure 1, [15]).

## Results and discussion

We have performed a large batch of simulations at and around the default values of the model parameters (Table 1). The actual parameter values are arbitrary, since no measurements are available to use them here, but they were chosen so as to reflect our best knowledge (or guess) regarding the physical-chemical properties of RNAs and their monomers. To our surprise the results proved to be very robust against changes of reasonable magnitude in almost all parameters anyway; only extreme values produced substantially different outcomes, and those were far out of the physico-chemically reasonable range.

**Table 1:** **Parameters of the model – ranges and defaults**

**Parameter**	**Value (boldface: default)**
grid size (*G*×*G*)	300x300
number of replicator diffusion steps (*D*)	¼ … 4 (**4**)
size of metabolic neighbourhood (*h*)	**3×3**,5×5,7×7 (Moore-type)
size of replication neighbourhood (*r*)	5 (von Neumann type)
number of different types of enzymes	3
maximum length of replicators (*L*)	70
claim of a site to remain empty, relative to the theoretical maximum of the sum of claims for an empty site (*ε*)	0.1
constant in Boltzann-distribution (*c*)	0.3
per base rate of substitution (*p*_sub_)	0.01
per base rate of deletion (*p*_del_)	0.001
per base rate of insertion (*p*_ins_)	0.001
scaling factor of replicability (*g*)	10
penalty factor of promiscuity (*σ*)	1.1
prefactor of (1-*p*_fold_) in replicability (*l*)	1
replication time (initiation) *b*_1_	1
elongation “length penalty” per base *b*_2_	0.001…0.5 (**0.05**)

The simulation study had two general focuses: the ecology (i.e., the coexistence criteria) and the evolution (i.e., the long-term equilibria of replicator sequence distribution and enzymatic functionality) of the metabolic replicator community. Obviously, evolutionary questions can be asked only in a system that is ecologically feasible, i.e., all the three metabolically cooperating, enzymatically active replicator types must be coexistent to be capable of evolution. We will show that the coexistence criteria of the present, physically more detailed model are in very good agreement with our toy model built earlier on similar, but largely implicit assumptions [15,18,19]. The ecologically relevant parameters of both the toy model and this one are the mobility of the replicators (*D*) and that of the small molecules (metabolites and monomers) represented by the size of metabolic neighbourhoods (*h*). Evolutionary changes in replicator structure and in metabolic functions thereof depend mainly on a single parameter related to the replication process: the per base elongation time (“length penalty”; *b*_2_). After having experimented with simulations varying all other parameters we concluded that the system is spectacularly insensitive to them, so we kept them constant at their default values, and systematically checked the effects of variation in *D*, *h* and *b*_2_.

The mineral surface (the lattice of 300 × 300 sites) was seeded in all simulations with a random population of replicators at an initial frequency of 80%, leaving 20% of the sites empty. The length of the initial replicators was also random with *L* evenly distributed on the [25,…,60] range. Simulations lasted for 10^6^ generations, after which the frequency of each replicator type (*F*_1_, *F*_2_, *F*_3_, *F*_12_, *F*_13_, *F*_23_, *F*_123_ and *F*_*p*_), the average of each enzymatic activity *A*_1_, *A*_2_ and *A*_3_ and the average length of each replicator type (*L*_1_, *L*_2_, *L*_3_, *L*_12_, *L*_13_, *L*_23_, *L*_123_ and *L*_*p*_) were recorded (index numbers correspond to different combinations of the three possible enzymatic activities as given in Figure 1; *p* stands for parasitic sequences). The ecological and the evolutionary aspects of the simulations are treated in separate sections below.

### Coexistence

**Figure 3:**
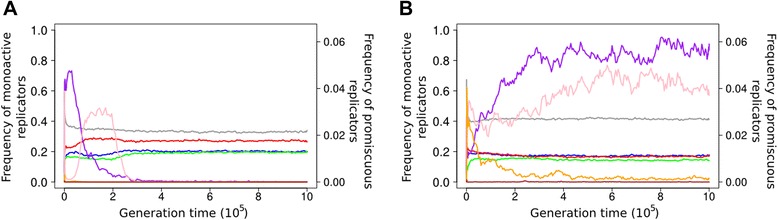
Time course of the frequency of different replicator types. Mono-active replicators with active centre {1} (blue), {2} (red), and {3} (green); bi-active replicators with two active centres {1,2} (purple), {1,3} (pink), and {2,3} (orange); replicators with all three types of active centre {1,2,3} (brown), and parasitic replicators without enzymatic activity {P} (grey). **A**: *D* = 4, *h* = 3x3, *b*_2_ = 0.05 (default parameter set). **B**: *D* = 4, *h* = 3x3, *b*_2_ = 0.001. Notice the different frequency scale (right vertical axis) for promiscuous enzymes!

In the random initial replicator population the proportion of enzymatically active replicators was low (slightly above 20%) with mono-active enzymes represented at the highest frequency, suggesting that enzymatically active secondary structures as defined by the rules of the model (Figure 3) are not very rare *per se* in a random sequence pool. Obviously, the emergence of two or more “active centres” within the same random sequence (i.e., the random occurrence of promiscuous enzymes) is much less likely but still occurs in 1-2% of the initial RNA pool. In most of the simulations the frequencies of mono-active replicators increase to their equilibria, while promiscuous enzymes almost completely disappear from the system after a temporary surge upwards (Figure 3A). This behaviour was qualitatively the same for all realistic parameter sets including the default. The frequency of functionless replicators (parasites) is also high in all simulations, and in good accordance with the original spatial MCRS model’s prediction [15]: cooperative replicators coexist with parasitic ones, and the latter cannot ruin the system. Figure 3B shows that the frequency of promiscuous enzymes depends on *b*_2_, the length penalty (elongation time) parameter: the smaller the penalty the longer the average replicator, which implies that it is easier to fit more than one active loops into its sequence. At the extremely low *b*_2_ = 0.001 the frequency of bi-active enzymes increases to about 10%, and even replicators with all three types of active loop remain in the system at a low abundance.

### Metabolic neighbourhood size *(h)*

Extremely small metabolic neighbourhoods are unlikely (or unable) to accommodate all the necessary enzyme activities and thus result in system collapse, because the number of catalytic activities is larger or just slightly smaller than the number of neighbourhood sites. This effect does not show up in our simulations, because system size (i.e., the number of different enzymatic activities for metabolism to work) is 3, whereas the smallest metabolic neighbourhood size used in the simulations is *h* = 3 × 3 = 9.

**Figure 4:**
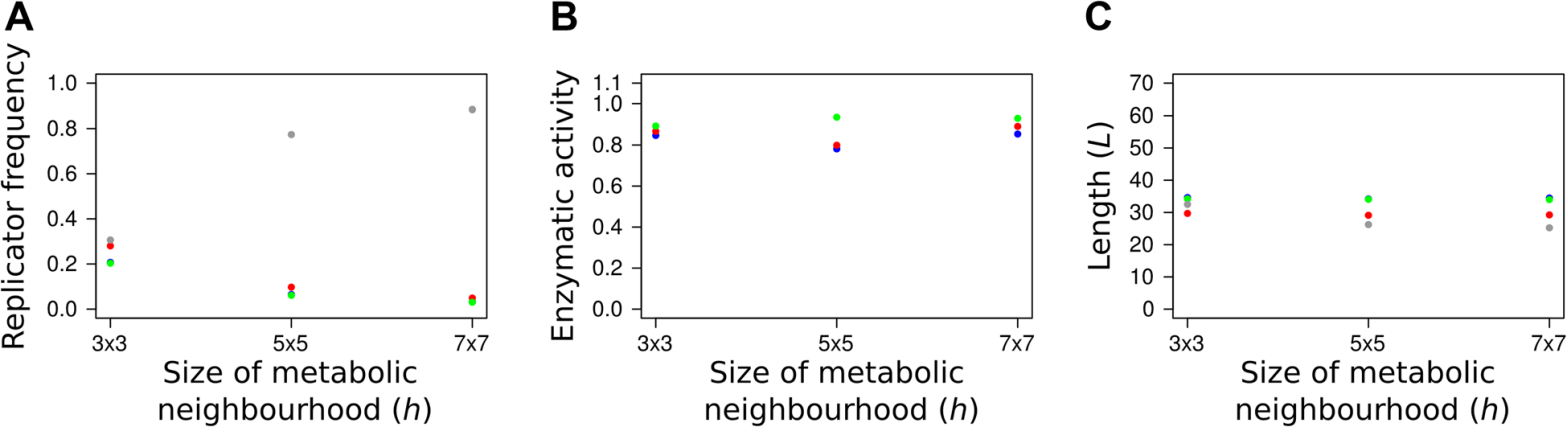
The effect of metabolic neighbourhood size on an evolving community of metabolically cooperating RNA replicators**.** The stationary frequencies of the different replicator types **(A)**, the averages of the different enzymatic activities **(B)**, and the average lengths of the different metabolic replicator types **(C)** in the stationary replicator populations after 10^6^ generations, at three different metabolic neighbourhood sizes (*3×3, 5×5, 7×7*). Replicators with enzymatic activity {1} (blue), {2} (red) {3} (green) and parasitic replicators without any enzymatic activity {P} (grey). Fixed parameters: *D* = 4, *b*_2_ = 0.05.

Figure 4 shows the effect of increasing metabolic neighbourhood size on stationary replicator frequencies and the evolved averages of enzyme activity and replicator length after 10^6^ generations. The frequencies of enzymatically active replicators (Figure 4A) decrease while parasitic replicators become more common with increasing metabolic neighbourhood size, suggesting that faster metabolite diffusion benefits parasitism. Indeed, if monomers are still available relatively far from where they were produced (i.e., far from the sites of enzymatically active replicators) then parasites can make use of their higher replicability *R* due to loose folding (small *p*_fold_ – check Eq. 5), and thereby take over in most localities. Increasing metabolic neighbourhood size represents a shift towards the mean-field version of the MCRS model which is known to go extinct for lack of advantage of rarity even without parasites [15,19]. Figures 4B and C show that the evolved enzymatic activities and the evolved lengths of the cooperating (metabolic) replicators do not depend on neighbourhood size. Note that at larger *h* values (5 × 5 and 7 × 7) parasites tend to be somewhat shorter than cooperating ribozymes, but the difference is not very large, even though length reduction is an efficient way to increase fitness through better replicability (Eq. 5). Decreasing length is obviously constrained in metabolic replicators, because they have to maintain their catalytically active structure, but this does not apply to parasites which are catalytically inactive. The fact that the parasites do not decrease their length to considerably shorter than that of the cooperators suggests that most of the surviving parasites might be the single- (or at most a few-) step mutant offspring of active metabolic replicators, with very small Hamming distances from the “masters”. Parasites constitute the non-functional mutant cloud (“quasispecies”, [27]) of metabolically cooperating replicators, and the genetic proximity of parasites to cooperators is maintained through mutation-selection balance. The shorter (faster replicable) mutants of the parasitic mutants are heavily selected against due to the disadvantage of aggressive parasites: wherever they become abundant they exclude their metabolically cooperating “hosts” locally and they perish for lack of replication resources (monomers). This mutation-selection balance represents high-turnover source-sink dynamics with respect to the replicator quasi-species.

### Replicator diffusion *(D)*

**Figure 5:**
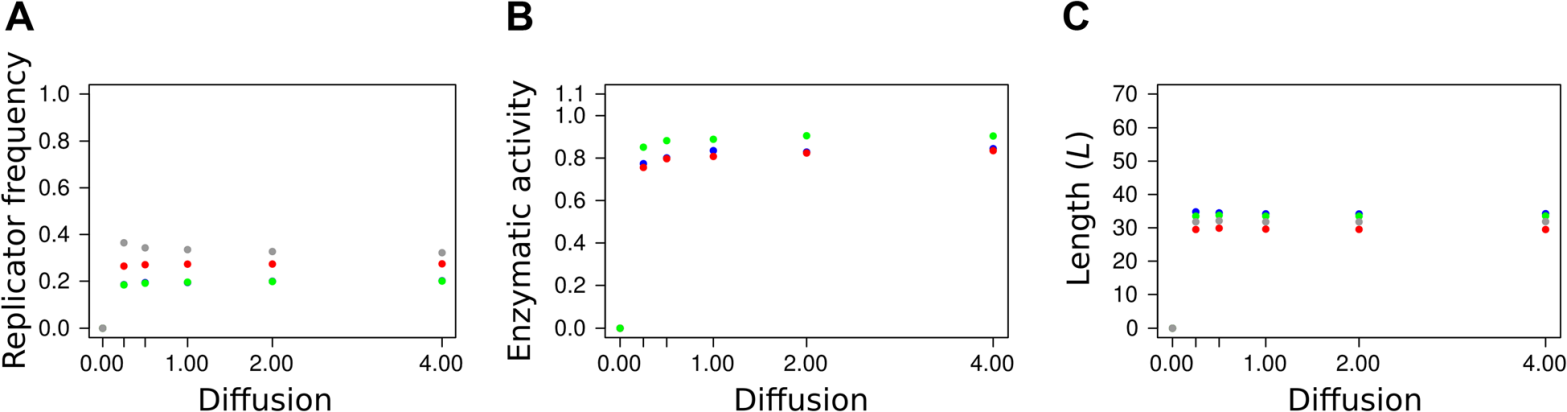
The effect of replicator mobility on an evolving community of metabolically cooperating RNA replicators The stationary frequencies of the different replicator types **(A)**, the averages of the different enzymatic activities **(B)**, and the average lengths of the different metabolic replicator types **(C)** in the stationary replicator populations after 3×10^6^ generations, at six different replicator mobilities (*D*) (0, 0.25, 0.5, 1, 2, 4). Colour code as in Figure 4. Fixed parameters: *h* = 3×3, *b*_2_ = 0.05.

Figure 5 shows that the replicator mobility parameter *D* acts in an all-or-none manner: zero diffusion kills the system, but almost any positive speed of diffusion is sufficient to maintain coexistence, and further increase in *D* does not change the results even in the quantitative sense. We have increased the diffusion parameter as *D* = 2^*n*^, *n*∈{−2,-1,0,1,2}, and found that none of the output variables – the stationary frequencies *F*_*i*_ (Figure 5A), the average enzyme activities *A*_*i*_ (Figure 5B) and the average length *L* (Figure 5C) – of the different replicator types had changed substantially within the range of *D* studied. With the overwhelming dominance of mono-active metabolic ribozymes this result is not very surprising: zero diffusion does not mix the different replicator types to the extent necessary for most metabolic neighbourhood to be complete, but even a very low threshold level of mixing may be sufficient for that, and it ensures long-term coexistence. Once it is above the threshold, increasing replicator mobility further does not change much in terms of metabolic efficiency and replicator coexistence. In fact even unrealistically high levels of replicator mixing – e.g., completely randomized spatial patterns – do not alter this result. Fast replicator diffusion does not help the parasite either, because it does not imply a shift of the model towards the mean-field – it is only the fast diffusion of metabolites (i.e., larger metabolic neighbourhood) that fosters parasite invasion, which is evident on Figure 4A. (Note that a metabolic neighbourhood size equal to lattice size is the limit at which the system comes at the mean-field.) Of course assuming fast replicator diffusion and slow metabolite diffusion at the same time would be very far from being physico-chemically realistic, but fortunately it is not necessary: very low replicator mobility is sufficient for the coexistence of the MCRS model. The case would be even more striking if promiscuous (bi- or tri-active) metabolic replicators were more abundant in the system, because then even less mixing would be sufficient for the average metabolic neighbourhood to be complete. Why is it then that enzyme promiscuity cannot be evolved or maintained even at extremely low replicator mobilities? For a system size of 3 (i.e., with metabolism requiring 3 different enzyme activities to be present within the same metabolic neighbourhood) there are two options to maintain complete local metabolisms with promiscuous replicators: 1) different bi-active replicators should be present at sufficient frequencies in a system still mixing somewhat to complement each other with their enzyme functions, or 2) tri-active replicators should prevail, in which case no mixing is needed at all. Both cases are obviously less advantageous than mono-active replicators in a slightly mixed system: 1) At any small *D* the mono-active replicators are at an advantage over bi-active ones, because promiscuous enzymes are less efficient in both catalytic functions than mono-actives (cf. Eq. 2 with *α*_*i*_ > 1), and bi-actives still need some mixing to avoid forming homogeneous patches on the surface and thus going extinct. Two of the three possible bi-active types ({1,2} and {1,3}) show up and then vanish during the first 3 x 10^5^ generations (Figure 3A), along with a temporary increase and subsequent decrease in average replicator lengths *L* (data not shown). This suggests that the initial difficulty of assembling three different mono-active replicators in the same metabolic neighbourhood is circumvented by evolving bi-active ones that are later excluded by their own, more efficient mono-active mutants. Note that {2,3} type bi-active replicators do not show up in large numbers because they need to be longer to be stable, and they are expendable because the two shorter bi-active types can deliver all the three necessary enzyme functions. 2) Tri-active replicators need to be much longer than mono-actives to fit all the three active loops into their secondary structure. This makes them much less probable to replicate than shorter replicators in the same replication neighbourhood, so that rarely emerging tri-active mutants are very likely fast eliminated from the system by their competing neighbours.

### Replicator sequence evolution

#### Folding energy

The Gibbs free energy (*E*) of a (folded) sequence defines the probability of the sequence being in the folded state (*p*_fold_, Eq. 1.). Replicability, enzymatic activity and replicator degradation rate are responsible for the dynamics of the system. These are the replicator traits that evolve, and all three of them depend on *p*_fold_ , albeit each one in a different way. Replicability and degradation rate (i.e., the *direct* components of replicator fitness affecting the rate of replicator birth and death, respectively, independently of their contribution to metabolism) are traded off: increasing *p*_fold_ decreases replicability (Eq. 5), which is a negative fitness effect, but it also decreases the rate of replicator degradation (Eq. 3), which is positive. The *indirect* fitness component is enzymatic activity, which increases with *p*_fold_ (Eq. 2) and improves the probability of replication through its positive effect on metabolism (Eq. 4). It is the combined fitness effect of these three fitness components at particular *p*_fold_ values (i.e., at certain folding energies) that is optimized during the evolution of the replicator community.

**Figure 6:**
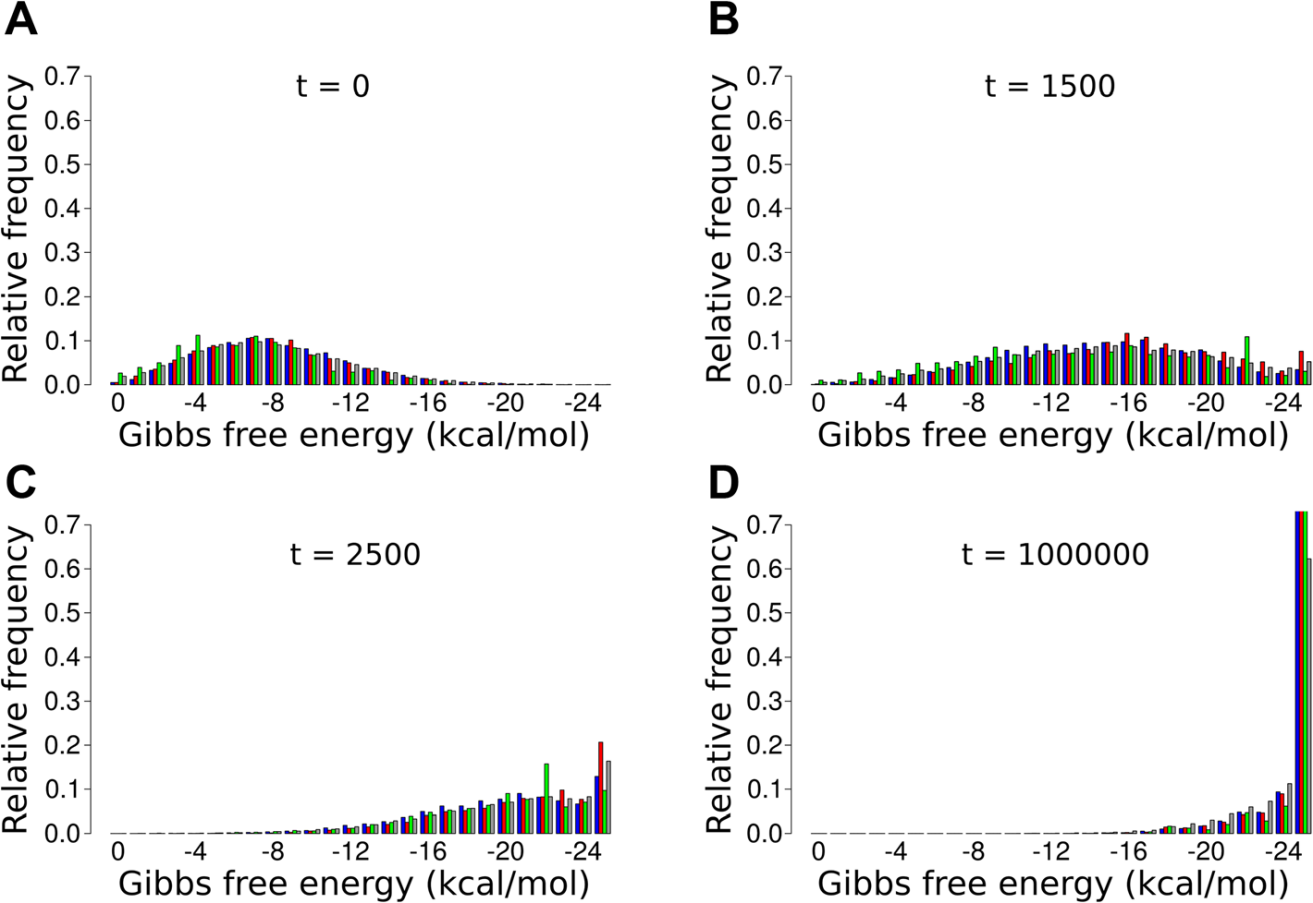
The evolution of the distribution of folding energy. Panels show the distribution of the folding energy of mono-active and functionless (parasitic) replicators at different times: **A**: *t* = 0, **B**: *t* = 1500, **C**: *t* = 2500 and **D**: *t* = 1000000. Colour code as in Figure 4. Parameters: *h* = 3×3, *b*_2_ = 0.05, D = 4.

Figure 6 illustrates how the mean of the folding energy distribution of the replicator community proceeds towards the low end of its possible range. The free energy histogram of the initial (random) population has a Cauchy-like profile (Figure 6A) for all replicator types. Note that enzymatic activity 3 (green on the histograms) requires the presence of the longest sequence-constrained loop in its secondary structure, thus it has the highest average folding energy of all replicator types; otherwise the profile of the distribution is the same as those of the other replicators. During the simulations the initial energy distribution flattens out at first (Figure 6B) and then the energy profile shifts towards the empirical energy minimum (*E*_min_ = −25 kcal/mol, Figure 6C) where, finally, it accumulates (Figure 6D) so that in the stationary replicator community most replicators belong to the lowest (optimal) energy class. Since a loop responsible for an enzyme activity can be embedded in sequences of very different folding energies, once a loop is in place the rest of the sequence evolves towards more durable (tightly folded) structures. Of course, to reach the energy minimum is not the direct target of the evolutionary process – the real target is the fitness maximum which, in this case, happens to coincide with the energy minimum (i.e., with tight folding). Compact folding is beneficial for fitness through its direct decreasing effect on replicator degradation and – for metabolic replicators – its indirect metabolic effect mediated by increased enzymatic activity. These advantages over-compensate its adverse effect on replicability.

The nearly identical energy distributions of functionless (*parasitic*) and metabolically active replicators at the stationary state of the system may be surprising at first, but the explanation is simple. Mono-active replicators easily mutate to parasitic ones, and parasites are in principle free to mutate further towards increasing their direct fitness (by higher replicability and/or lower degradation). However, fast replicating parasites are strongly selected against locally, because they demolish metabolism in their own vicinities and starve to death. That is, parasites are quickly eliminated by local selection, and permanently reintroduced by mutation from metabolically active replicators. Thus the parasites we see in the system are the close sequence relatives of functional metabolic enzymes, representing their parasitic quasispecies [27] created by mutation and effectively selected against on a peaky fitness landscape. Sequence similarity implies energetic similarity on average – this is why the energy profile of parasites remains close to that of metabolic replicators.

### Length penalty

To understand the effect of the length penalty parameter (*b*_2_) we have to see that the system maintains a delicate evolutionary balance between high replicability (which implies short sequences, Eq. 5) and efficient enzymatic activity (which requires long enough sequences) Eq. 2). The replicability criterion and the enzymatic efficiency criterion act on the length of the sequence (*L*) antagonistically, resulting in a *trade-off* relation between these evolving traits. On the one hand, the shorter the sequence the faster its replication. On the other hand, longer sequences may acquire more complex folded structures which in turn may account for a more efficient enzymatic activity. Besides the length of the sequence, enzymatic efficiency depends on the energy of the folded structure, too, but folding energy may change with sequence changes almost independently of *L*. The actual length of the evolved replicators (around 30 nt, which is, amazingly, almost independent of model parameters) is the optimum compromise between all the length-dependent fitness criteria.

**Figure 7:**
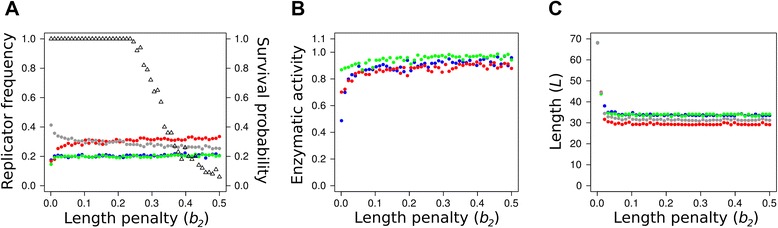
The effect of the length penalty (*b*_2_) on replicator evolution. **A**: the stationary frequencies of replicator types (triangles: the proportion of surviving systems out of 100 parallel simulations); **B**: the averages of available enzymatic activities; **C**: the average length of replicator types. Data points recorded at generation *T* = 10^6^. Colour code as in Figure 4. Fixed parameters: *h* = 3×3, *D* = 4.

Recall that the length penalty parameter represents the time needed to add a single nucleotide to a new replicator being produced during the template copy process. Low length penalty means that the elongation of a long sequence is punished less (in terms of the replicability component of fitness) and consequently the length of replicators evolves to the upper limit allowed in the model (70 nt, Figure 5C). The increase of stationary replicator length at extremely low length penalty is driven by the fitness advantage (the decrease of degradation rate and the increase of enzyme activities) due to the more compact structure that longer sequences can acquire. Longer replicators can accommodate multiple active centres more easily – this is why promiscuous ribozymes can reach much higher proportions at the extremely small, biologically unrealistic length penalty *b*_2_ = 0.001 (cf. Figure 3). This effect also shows up on Figure 7B, where the relatively small enzyme activity of mono-active replicators within the 0 < *b*_2_ < 0.05 range is due to the fact that some of the activity is in promiscuous sequences which are not included in the data. The frequency of promiscuous enzymes is negligible above this range.

Even a very small increase of *b*_2_ (i.e., a small decrease in the speed of replication) is sufficient to cut back the stationary length of the replicators to 30-35 nt, and it essentially does not change with increasing length penalty any further (Figure 7C). This suggests that 30-35 nt is about the minimum size of a replicator capable of maintaining a single enzyme activity in a sufficiently stable structure. Since the parasitic replicators present in the system are the products of a single (or at most a few) point mutations of mono-active metabolic replicators, they preserve the length of their masters, as explained in the previous section.

The most conspicuous effect of variation in length penalty is on the ecology of the metabolic replicator community, which is summarized in Figure 7A. The triangles on the graph represent the proportions of surviving systems out of 100 independent simulations, each initialized with different random number seeds, but identical otherwise. The coloured data points show the actual stationary frequencies of the different replicator types after 10^6^ generations, provided that the system survived. Within the *b*_2_ ~ [0–0.25] range the replicator community reaches the stationary state in all simulations, with the mean frequency of replicators (averaged over the 100 replicate simulations) falling between 0.2 and 0.35. These stationary frequencies do not change at higher length penalties either, but from *b*_2_ = 0.25 upwards the metabolic replicator community may collapse, and it does so with increasing probability. That is, the system either survives and approaches its intrinsic equilibrium or it dies out; the probability of extinction increases with the length penalty parameter. *b*_2_ = 0.25 may be the break-even point for the average initial fitness of the system: below 0.25 the combined effect of the three fitness components (replicability, degradation rate and enzyme activity) results in positive initial population growth potential on average, which leaves ample possibility for replicators to evolve towards even higher fitness – as a result the replicator community always coexists and evolves under *b*_2_ = 0.25. Increasing the length penalty decreases the average replicability of the replicators (cf. Eq. 5), implying that their average reproductive potential decreases (Eqs. 6 and 7). At about *b*_2_ = 0.25 the average growth potential of the initial (random) replicator community turns to negative, so that at length penalty above 0.25 the average initial system starts to decrease. The shrinking replicator community cannot escape going extinct unless a lucky combination of the fitness components creates a local “seed” of metabolic replicators with positive growth potential, which may then invade the whole lattice. The booting up of surviving systems starts with the temporary proliferation of bi-active ({1,2} and {1,3}) replicator types capable of performing all the three necessary catalytic functions together (Figure 4A), which in turn requires a temporary increase in replicator length *L* (data not shown). The chance for this to happen decreases with increasing *b*_2_, because high length penalty implies a fast track to extinction, with little chance for the promiscuous “seed” to assemble. The evolved community consists of all the three metabolic replicators accommodating at least one enzymatic activity each, so their average stationary length cannot go below the empirical threshold *L* ~ 35 (Figure 7C).

## Conclusion

Perhaps the most striking feature of the model is its remarkable robustness against changes in almost any of its parameters. The two exceptions are metabolic neighbourhood size (*h*) and the cost of replicator elongation (length penalty; *b*_2_), but even those act only on the ecology of the replicator community, leaving the evolved traits of the replicators (length, folding energy, enzyme activity) mostly unchanged.

### Coexistence

Metabolic neighbourhood size defines the distance of the system from the mean-field case in which all the replicators have the same monomer supply and thus the one of highest replicability inevitably excludes all the other ones and the metabolic system collapses. Small *h* gives an advantage to metabolically active replicators, and the advantage is highest for the least abundant metabolic replicator type. This advantage of rarity keeps the replicator community coexistent, but increasing *h* benefits parasitic replicators which become abundant in the stationary replicator community at larger metabolic neighbourhoods (Figure 4A). The conclusion from all this is that for an efficient metabolic replicator system to be maintained a small metabolic neighbourhood size is necessary, which entails three different physical-chemical properties of small metabolites and monomers: slow surface diffusion and/or fast degradation and/or fast desorption from the surface. The direct criterion of coexistence is that metabolites and monomers do not drift far from the enzyme producing them before they are a) used in a metabolic reaction (metabolites) or in replication (monomers), b) degraded, or c) desorbed from the surface. Even though increasing *h* decreases the stationary frequencies of metabolic replicators and increases that of parasites, it does not affect the evolved traits of metabolic replicators: they remain essentially the same across the range of *h* studied (Figure 4B and C). The length penalty parameter (*b*_2_) affects system survival in a probabilistic manner: the higher the length penalty the less chance for the metabolic replicator community to survive (Figure 7A), but the evolved features of surviving systems are also the same across the *b*_2_ range.

### Robustness

Another conspicuous feature of the data on Figures 3, 5, 6 and 7 is that the evolved enzyme activities, folding energies and lengths of the different replicator types are not only invariant across the relevant parameter ranges of the model, but they also seem to converge: at any specific value of *D*, *h* or *b*_2_ the data points representing the different replicator types are very close to each other. This suggests that the evolving traits (length, folding energy, enzyme activity) of the replicators tend to become similar during the evolution of the communities, and this tendency to trait convergence is essentially independent of the actual system parameters. The underlying principle might be that of *group selection* [28]. The most efficient local communities are those in which all the three metabolically active replicators are present in a uniform frequency distribution, because these produce the most monomers to support their own reproduction (cf. Eq. 4). Maintaining the optimal even distribution of metabolic replicators in the long run – and thus the highest indirect fitness for the community – is possible only if the fitnesses of the replicators are as similar as possible, hence the group selection pressure towards uniform replicator traits. Note that local group selection is also the ultimate mechanism that keeps the system coexistent in the first place – its effect through evolved fitness evenness is an extra component of ecological robustness in the Metabolically Coupled Replicator System.

### Enzyme efficiency and specificity

We have addressed two general aspects of ribozyme evolution with this model: those of *efficiency* and of *specificity*. Adaptation in efficiency means improvement in the activity of the enzyme in the reaction it is supposed to catalyse. Enzyme activity is dependent on the *substrate affinity* and the *conversion efficiency* of the active centre. These are traits which can evolve somewhat independently of each other, the first being dependent on the size and shape of the binding pocket [29], the second on the positions of chemically active groups inside the pocket. Of course the simultaneous evolution of substrate affinity and conversion efficiency is well possible [30], as implicitly assumed in the different enzymatic activities attributed to different active site sequence patterns (Figure 2) in the model. The fact that mean ribozyme activity jumps close to its theoretical maximum from the low activity start in a very short time and it remains high for any parameter setting shows that the indirect (metabolically mediated) replicator fitness component is under very strong positive selection.

It is probable (and, for a successful launch of the evolution of catalysis, also sufficient) that early ribozymes were not very efficient, but they might have possessed more than a single catalytic activity (*enzyme promiscuity*). Catalytic promiscuity is known to exist both in protein and in RNA enzymes, with different mechanisms in the two cases. For protein enzymes, the binding pocket may either attach different substrates (*substrate promiscuity*), and/or it can catalyse more than a single reaction on the same substrate (*catalytic promiscuity*). In RNA enzymes the mechanism of promiscuity is different, because it occurs through different foldings of the same sequence, so that the same ribozyme can operate as two different enzymes in time-sharing mode [31-33].

The evolutionary advantages of temporary ribozyme promiscuity are obvious, if different catalytic functions need to be present at the same location for a vital function of an evolvable system to work, even if the cost of catalytic promiscuity is paid in reduced enzyme activity, as is the case for ribozymes in general. Keeping different, functionally complementary ribozymes together can be realized through closing them in vesicles [34] or by constraining their mobility on mineral surfaces [15,35,36]. In either case, communities of promiscuous ribozymes may increase against the odds of extinction due to local demographic stochasticity when catalytically active replicators are scarce – i.e., in the initial state dominated by random, mostly catalytically inactive sequences. However, for promiscuity to persist in spite of the higher efficiency of specialized catalysts, replicator mobility needs to be extremely low [18] – which is a condition rather unlikely to occur both in vesicles and on mineral surfaces. For metabolisms of even a few more than two reactions the promiscuity of the enzymes gives only a marginal advantage, because bi-active ribozymes still need to be complemented by other ones, which requires replicator mobility anyway. Based on our model’s predictions it seems likely that metabolically more efficient mono-specific ribozymes had soon taken over the catalytic functions of promiscuous ones after a short transient upshot of the latter. The evolutionary process of ribozyme specialization might have proceeded through the effects of the replicators’ fitness components (degradation rate, enzymatic activity, replicability) which are all mediated by the folding probability *p*_fold_ and, ultimately, by the folding energy *E* of the replicator sequences.

In previous MCRS models [15,17,19] we have implemented replicators as abstract chemical entities capable of self-reproduction and enzyme activity. Those studies were aimed at verifying the MCRS concept as a feasible mechanism for prebiotic replicator coexistence. The present model differs from the previous ones in that the abstract replicators are replaced by RNA sequences, the energetic features and the corresponding 2D folding structures of which are explicitly considered in defining their enzymatic activities, decay rates and replicabilities. The aim of this study was to demonstrate that the MCRS concept does work with RNA sequences featuring realistic physicochemical properties (2D structure and free energy) at least as well as it does with abstract replicators. Our results suggest that the MCRS principle is as robust in this model as it was in the previous ones, leading to dynamics qualitatively similar to those of the toy models: the system of metabolic replicators stays coexistent indefinitely, and it resists destructive parasitism by fast replicating, non-enzymatic RNA sequences. An additional, attractive feature of the new model is its remarkable insensitivity to changing its crucial parameters across wide ranges, the likely reason of which might be the feedbacks and trade-offs realized through the folding energetics of the sequences – i.e., more realistic replicator traits that the toy models did not consider. The consequent improved structural stability of the explicit replicator system lends further support to the fundamental logic of the MCRS concept.

We have also addressed the question of possible evolutionary scenarios for building up MCRS in previous work [18] using abstract replicators. The same problem is, of course, pertinent with explicit RNA replicators as well. The occurrence of new metabolic enzyme activities and the increase of existent ones in mutant replicators is a possibility in the present model as well as in the toy versions, the only substantial difference between the two approaches being the effect of diffusion on the evolution and the dispersion of promiscuous replicators admitting multiple enzyme activities: it was enhanced by increasing diffusibility in the toy model, whereas it remained in essence the same in the explicit version.

## Reviewers’ report

### Reviewer 1: Gáspár Jékely

In this paper Könnyű and colleagues extend their Metabolically Coupled Replicator System (MCRS) to simulate the early evolution of metabolically coupled ribozymes on a mineral surface. The main novelty in their approach is the explicit calculation of RNA secondary structures and minimal free energies for the evolving sequences and the use of the calculated parameters for the model simulations. This makes the model more realistic than the earlier versions. I suggest that the authors state more explicitly what are the new insights gained from using the more realistic secondary structure and free energy calculations. For example the authors observe that: “The shorter (faster replicable) mutants of the parasitic mutants are heavily selected against due to the disadvantage of aggressive parasites”. Was this already shown in the less explicit versions of the MCRS model? An extra paragraph in the Conclusions section could summarize the novel results.

*Two extra paragraphs have been added to Conclusions section summarizing the novelties of the results.*

It would be interesting to see results with larger metabolic neighbourhood (h = 7×7) and higher replicator diffusion. One would expect that parasitic sequences can diverge more since they will not as easily demolish metabolism in their own vicinities, due to larger diffusivity.

*The results of the simulations with h = 7×7 and D = 4 (Figure**3**) show that increasing metabolic neighbourhood size, even if it decreases replicator frequency, does not affect the stable persistence of the system. Its stationary characteristics (the equilibrium distributions of replicator frequencies, enzymatic activities and sequence chain lengths) have also been largely insensitive to the actual value of the dispersal parameter D (Figure**4**). This is in good accordance with our previous results [19] obtained with toy model simulations scanning a wide range of the (D, h, r) parameter space. The earlier study revealed a positive effect of increasing dispersal (D) which prevents the aggregation of conspecific replicators, and a negative effect of increasing h, though approximating the mean-field situation that is known to go extinct. The deleterious effect of larger h can be compensated by increasing D to some extent, but the compensatory effect is limited: at too large metabolic neighbourhood sizes the system collapses anyway (cf. [19]: Figures**2**and**4**). The parameter setting suggested by the Reviewer (large h, large D) is, unfortunately, practically not feasible in the explicit model, because even on a high-capacity grid computer it would take months to run a single simulation. The huge difference between the CPU time demands of the toy models and the present one is due to the “handling time” of sequence folding. However, the results of the toy models are likely to carry over to the explicit case in this respect, too, since in all other respects we experienced qualitative matches.*

Importantly, the authors should provide their code as an Additional file or deposit it to a public repository for others to reproduce or extend the model calculations.

*As the code is the result of a long process of development, and it will be further developed for later studies, we do not find it convenient to publish it at this stage. However, we are willing to send the code to the reviewer or to anyone for further studies or for reproducing our results, on an individual basis.*

Minor comments:

pg 19–20 The discussion about the dynamics of parasites is repetitive in this section. I suggest to delete or shorten this part: “Mono-active replicators easily mutate to parasitic ones … their own vicinities and starve to death”.

*We have shortened this part.*

Typos:

page 11: “we assume that different RNS molecules” change to RNA

*Corrected.*

page 17: “i.e., larger metabolic neighbourhood) that fosters parasite invasion, which is evident on Figure 4A”. - change to Figure 3A

*Corrected.*

### Reviewer 2: Anthony Poole

This is a very elegant study which has been explained in very accessible language. I found the results very insightful, and need say little other than that, for those interested in the RNA world, this is a paper well worth reading.

*We thank the Reviewer for their positive judgment of the study.*

For my money, the most exciting result is that this model suggests that catalytic promiscuity in early ribozymes may have been extremely short-lived. This bears thinking about, particularly given the view, popular in protein science circles that early enzymes were promiscuous (both in their substrate specificity and enzymatic reactions). It is also noteworthy that group selection appears as a feature of the model. This is broadly consistent with the cooperative networks that Lehman and colleagues observed for fragmented ribozymes (Nature 491:72–77). I would be interested to see a brief discussion of that work and how it relates to the authors’ findings.

*Lehman and co-workers showed that mixtures of RNA fragments that self-assemble into self-replicating ribozymes can form catalytic cycles and more complex networks. We agree that some aspects of our model show some similarity to some of Lehman’s, even though the two models (Lehman’s and ours) are essentially different in their basic assumptions. Ours assumes cooperation in the evolutionary sense, so that a complete metabolic neighbourhood (a cooperating “team” of potentially competing replicators) is capable of replicating a focal sequence which thus will have two identical copies locally (apart from mutations). The model of Lehman, on the other hand, assumes collective autocatalysis: in which the complex networks arise due to the fact that the members of the set catalyze each other’s formation, rather than replication. Yet, it is true that both models are prone to being parasitized and ultimately exterminated by parasitic replicators in a mean-field framework, both requiring some form of spatial structure as a potential defense: “Longer term evolutionary optimization would have required spatial heterogenity or compartmentalization to provide lasting immunity against parasitic species or short autocatalytic cycles.” (Vaidya et al. Nature 491:77 (2012)).*

Just a minor quibble about this statement in the Background: “recent organisms still carry reliable clues suggesting that RNA had played a central role both in the metabolism and in the genetics of very early forms of life [3,4]”. The papers cited here are both excellent, but address the more chemical aspects of the origin of RNA itself and of RNA catalysis. By contrast, the recent paper by Hoeppner et al. (PLoS Comp Biol 8: e1002752. http://dx.doi.org/10.1371/journal.pcbi.1002752) used a comparative genomic approach to look at whether there are ‘clues’ of the RNA world in modern organisms, so is perhaps more appropriate, given the sentence.

*Thanks for drawing our attention to the paper – we have cited it in the corresponding part of the text.*

Minor comments/typos (can be deleted from the review once addressed):

Page 7, the use of the term “catching” - perhaps “binding” is more appropriate here. Enzymologists routinely talk about substrate binding and product release.

*We rephrased the sentence in a more clear way.*

Page 11, “hydroxil” should be hydroxyl

*Corrected.*

P11, RNS should be RNA

*Corrected.*

P26, “thedesing” should be the design

*Corrected.*

Figure 2 X-axes: Ferquency

*Corrected.*

### Reviewer 3: Armen Mulkidjanian

Könnyű and co-workers have attempted to fill the gap between purely mathematical, "toy" modeling of very early evolution and the physico-chemical realm within which such evolution may have proceeded. Specifically, the model of Könnyű and co-workers explicitly accounts for the primary and secondary structure of replicators. Hopefully, the authors would continue their efforts to model the physics and chemistry of the early evolution. Therefore, the comments below contain certain recommendations which could be realized either upon revising of the given manuscript or in the future work of the authors.

Major comment:

1) My major concern is the plausibility of the metabolic part of the model. The authors assumed that “the different RNA species cooperate to produce monomers for their own replication, and possibly also to supply other “common goods” for the replicator community”. Thereby, only three catalytic activities were assumed to be sufficient to perform all these functions in the model of the authors. Obviously, the assumption of only three catalytic activities is an oversimplification, which is quite understandable in the given context. In a wider context, however, the source of monomers and “common goods” for the first replicators is one of the open questions in the origin of life research. Apparently much more than three catalytic activities should have been simultaneously needed to produce different monomers and, in addition, the “common goods”. Furthermore, there is no evidence of ribozymes capable of synthesizing nucleotides from scratch; the chemistry of the RNA catalysis, as revealed so far, is not very encouraging in this respect. A possible solution for this conundrum is that monomers and other common goods (e.g. amino acids and sugars) could be initially produced in abiotic reactions [1–5]. Only later, step by step, the first replicating entities may have learned to synthesize nucleotides. Syntheses of nucleobases and amino acids were recently demonstrated in the solutions that contained formamide or urea and in the presence of UV-light, see [6,7] for reviews. Environments with high levels of amides/urea could be envisioned on the primordial Earth [6,8,9]. The hypothesis of abiotic origin of monomers got a major boost after Sutherland, Powner and their co-workers succeeded in synthesizing nucleotides from scratch in geologically plausible, one-pot settings [10,11]. In addition, these authors have shown that natural nucleotides were particularly UV-stable, so that their relative fraction selectively increased under UV illumination [10], in support of earlier theoretical predictions [12]. In such a case, the very first replicators would require only catalytic abilities of assembling abiotically formed monomers - a task that should have been feasible for ribozymes. Hence, scenarios of “abiotic syntheses” match the simplified approach of Könnyű and co-workers in that they imply only few catalytic activities. In a framework of such scenarios, however, an essential source of monomers for replication would be the decay of other replicators. Furthermore, the abilities to accelerate this decay by cleaving neighboring sequences (which would correspond to a reversion of the assembly reaction) as well as to use the fragments for building the own “body” would be extremely advantageous. A scenario of “abiotic syntheses”, of course, would require a separate model that would differ from the model in the given manuscript. Still, in the view of anticipated importance of replicator decay/cleavage, an analysis of the present model in relation to the decay processes, namely a consideration of the model outcome as a function of decay parameters (currently absent from the manuscript) might be of use for readers.

*We absolutely agree with the Reviewer in that even a very simple realistic metabolism requires a lot more different types of catalysts than postulated in our model. However, we are also sure that the only way such a metabolically competent ribozyme set could have evolved is through the retroevolutionary mechanism explained in some detail elsewhere [20–21], which must have started from abiotiocally produced monomers at its very beginning, exactly as the Reviewer suggests. We have inserted a paragraph into the Background section pointing this out explicitly and citing the relevant literature.*

Minor comments:

2) The manuscript would benefit from a graphical presentation of the focal cell with its neighborhood. Without such a figure, the sentence “For a replication event to occur the focal replicator *s* must be complemented by all three different enzymatically active molecules in its metabolic neighborhood (MET(*h,s*), the set of *h* sites concentric on the site of the focal replicator *s*)” is not quite clear.

*We added a figure that explains metabolic and replicator neighbourhood configurations.*

3) It is not clear how the model accounts for “the time consumption of “releasing” the product and “catching” the next substrate for catalysis”. The whole section on sub-additive effects is rather incomprehensible and showed be re-written in a more clear way.

*We rephreased it in a more clear way.*

4) The statement that "replication is possible only in the unfolded state" should be defined as one of assumptions of the model. Generally, it is possible to imagine that unfolding could proceed concurrently with replication. That is how replication occurs in our cells.

*Indeed, we cannot exclude the possibility of the simultaneous unfolding and replication of RNA molecules on the basis of first principles, but note that our postulate of the temporal separation of the two processes is in fact a worst-case assumption: the chance of catalytically active, low-energy folds to be replicated is small. Therefore if this assumption has any effect on the results, then it is negative, but we actually think that changing it may not alter the results in the qualitative sense.*

References

[1] A.I. Oparin, The Origin of Life, Moskowskiy rabochiy, Moscow, 1924.

[2] J.B.S. Haldane, The Origin of Life, in: C.A. Watts (Ed.) The Rationalist Annual 1929, pp. 3–10.

[3] N.H. Horowitz, On the Evolution of Biochemical Syntheses, Proc. Natl. Acad. Sci. USA, 31 (1945) 153–157.

[4] S.L. Miller, L.E. Orgel, The Origins of Life, Prentice Hall Englewood Cliffs, NJ, 1973.

[5] A. Lazcano, S.L. Miller, On the origin of metabolic pathways, J. Mol. Evol., 49 (1999) 424–431.

[6] S.A. Benner, H.J. Kim, M.A. Carrigan, Asphalt, water, and the prebiotic synthesis of ribose, ribonucleosides, and RNA, Acc Chem Res, 45 (2012) 2025–2034.

[7] R. Saladino, C. Crestini, S. Pino, G. Costanzo, E. Di Mauro, Formamide and the origin of life, Phys Life Rev, 9 (2012) 84–104.

[8] A.Y. Mulkidjanian, A.Y. Bychkov, D.V. Dibrova, M.Y. Galperin, E.V. Koonin, Origin of first cells at terrestrial, anoxic geothermal fields, Proc Natl Acad Sci U S A, 109 (2012) E821-830.

[9] D.V. Dibrova, M.Y. Chudetsky, M.Y. Galperin, E.V. Koonin, A.Y. Mulkidjanian, The role of energy in the emergence of biology from chemistry, Orig Life Evol Biosph, 42 (2012) 459–468.

[10] M.W. Powner, B. Gerland, J.D. Sutherland, Synthesis of activated pyrimidine ribonucleotides in prebiotically plausible conditions, Nature, 459 (2009) 239–242.

[11] M.W. Powner, J.D. Sutherland, J.W. Szostak, Chemoselective multicomponent one-pot assembly of purine precursors in water, J Am Chem Soc, 132 (2010) 16677–16688.

[12] A.Y. Mulkidjanian, D.A. Cherepanov, M.Y. Galperin, Survival of the fittest before the beginning of life: selection of the first oligonucleotide-like polymers by UV light, BMC Evol Biol, 3 (2003) 12.

## Abbreviation

MCRS: Metabolically Coupled Replicator System
